# Supplementary material for: Subclinical Atherosclerosis Is Associated with Discrepancies in BAFF and APRIL Levels and Altered Breg Potential of Precursor-like Marginal Zone B-Cells in Long-Term HIV Treated Individuals
Source: Vaccines (Basel). 2022 Dec 30;11(1):81. doi: 10.3390/vaccines11010081 (PMC9863280; doi:10.3390/vaccines11010081)
Supplement: Supplementary file 1 [file vaccines-11-00081-s001.zip › vaccines-2089372-supplementary.pdf]

Supplemental Table S1 : Sociodemographic characteristics of the sub-cohort (B) used in this study

Group	HIV- CVD- (n=10)	HIV- CVD+ (n=20)	HIV+ CVD- (n=18)	HIV+ CVD+ (n=39)	p-value
Age	52.93 (39.2 – 62.19)	55.29 (42.79 – 70.57)	51.77 (40.61 – 70.13)	55.23 (44.22 – 74.43)	0.28
No. of male participants[1]	NA	NA	NA	NA	NA
TPV (mm^3)	0	371.7 (34.0 – 1948.0)	0	435.4 (6.74 – 1981)	0.49
LDL (mmol/L)	3.54 (2.36 – 4.52)	2.88 (1.67 – 5.44)	2.99 (1.92 – 4.23)	2.70 (1.15 – 4.57)	0.051 (*)[2]
HDL (mmol/L)	1.33 (0.73 – 2.92)	1.28 (0.89 – 1.99)	1.29 (0.72 – 2.34)	1.26 (0.75 – 2.57)	0.9414
Median 10 years Framingham Risk Score (%)	12.2 (2.00 – 18.00)	11.89 (5.00 – 18.00)	8.44 (4.00 – 18.00)	10.23 (3.00 – 27.00)	0.069
Participants undergoing statin therapy (%)	1 (10%)	4 (25%)	3 (17.64%)	14 (35.9%)	0.22

[1]All participants are male in this cohort

[2]Significant differences between HIV- CVD- and HIV+ CVD+ (p = 0.04), as assessed by the Kruskal-Wallis test with post-hoc Dunn's.

Supplemental Figure S1: Gating strategy for flow cytometry analyses. After excluding doublets and dead cells, total B-cells (CD19+) were positively gated. Then, CD1c+ cells were selected, followed by IgM^{+} $CD27^{+}$ double-positive cells. From the latter, the CD10- population was designated as mature marginal zone (MZ) B-cells, while the CD10+ population was designated as MZ precursors (MZp).

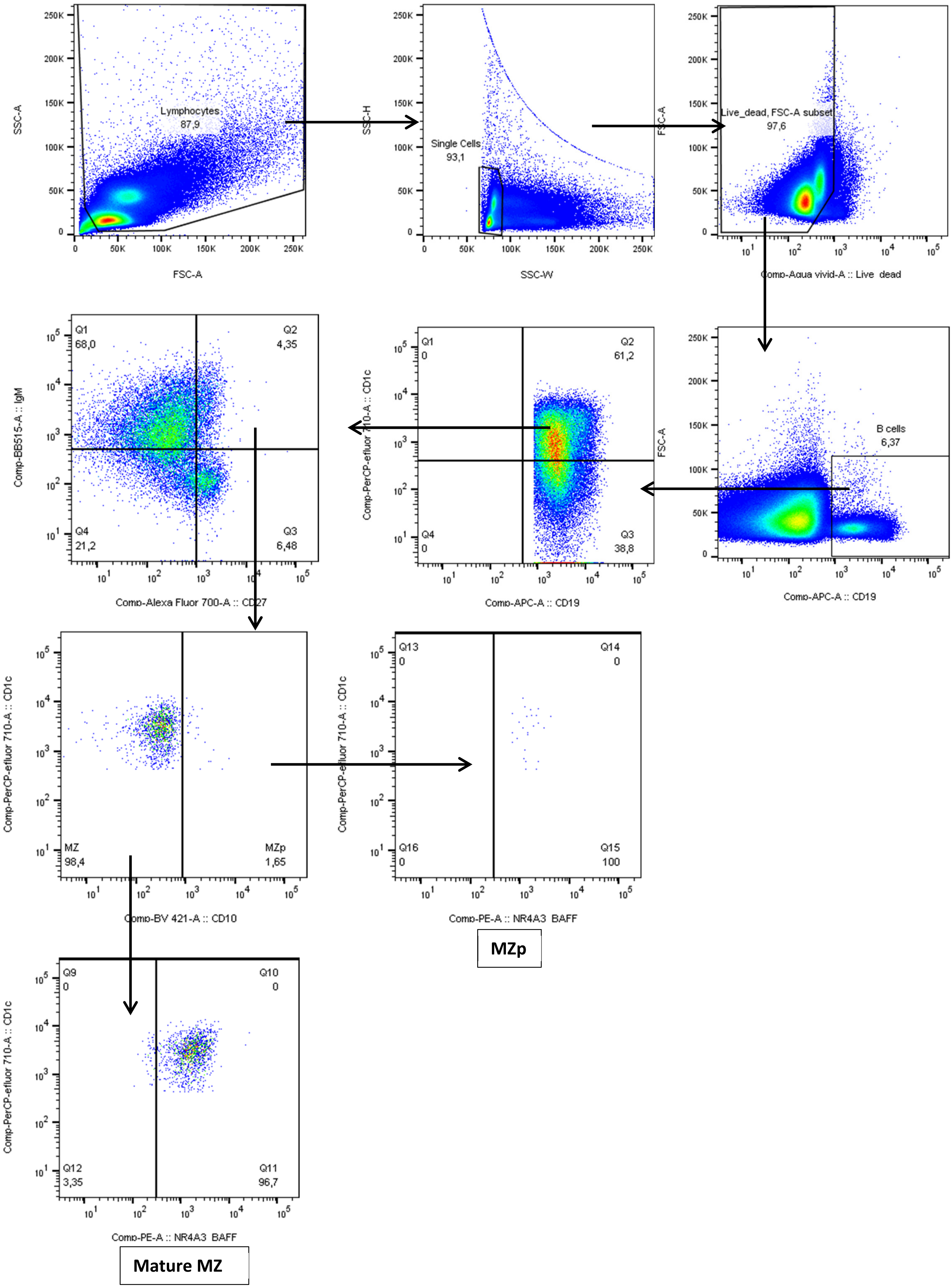

SSC-A
FSC-A
Lymphocytes
87,9
SSC-H
SSC-W
Single Cells
93,1
FSC-A
Live_dead, FSC-A subset
97,6
Comp-Aqua vivid-A :: Live_dead
Comp-BB515-A :: IgM
Q1
68,0
Q2
4,35
Q4
21,2
Q3
6,48
Comp-Alexa Fluor 700-A :: CD27
Comp-PerCP-efluor 710-A :: CD1c
Q1
0
Q2
61,2
Q4
0
Q3
38,8
Comp-APC-A :: CD19
FSC-A
B cells
6,37
Comp-APC-A :: CD19
Comp-PerCP-efluor 710-A :: CD1c
MZ
98,4
MZp
1,65
Comp-BV 421-A :: CD10
Comp-PerCP-efluor 710-A :: CD1c
Q13
0
Q14
0
Q16
0
Q15
100
Comp-PE-A :: NR4A3_BAFF
MZp
Comp-PerCP-efluor 710-A :: CD1c
Q9
0
Q10
0
Q12
3,35
Q11
96,7
Comp-PE-A :: NR4A3_BAFF
Mature MZ

Supplemental Figure S2: BAFF levels and correlations between soluble BAFF and total plaque volume in the blood of HIV-uninfected and HIV-infected individuals of the CHACS sub-cohort (B). Levels of soluble BAFF (A) in the blood of HIV uninfected participants, without and with CVD (HIV-CVD-, HIV-CVD+, respectively), and HIV-infected participants, without and with CVD (HIV+CVD-, HIV+CVD+, respectively) selected in sub-cohort (B). Correlation between soluble BAFF and total plaque volume in HIV+ CVD+ (B) and HIV- CVD+ (C) participants of the sub-cohort (B). Normality was assessed with the Shapiro-Wilk test. A Kruskal-Wallis test with a post-hoc Dunn's was used for testing statistical differences between groups in Supp. Fig. 1A. A Spearman correlation was used for testing for the correlations in Supp. Fig. 2B, C. CVD – Cardiovascular Diseases. * $p < 0,05$; ** $p< 0,01$; *** $p< 0,001$; **** $p < 0,0001$

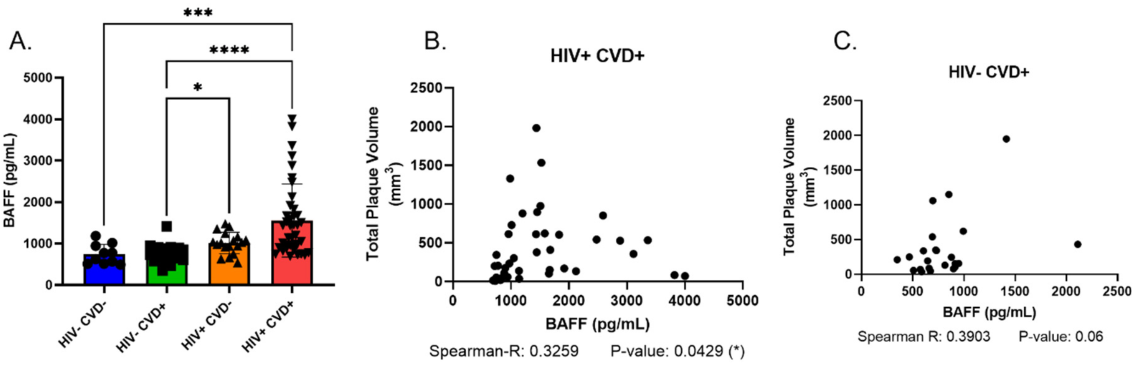
A.
***
****
*
BAFF (pg/mL)
5000
4000
3000
2000
1000
0
HIV- CVD-
HIV- CVD+
HIV+ CVD-
HIV+ CVD+
B.
HIV+ CVD+
Total Plaque Volume (mm3)
2500
2000
1500
1000
500
0
0
1000
2000
3000
4000
5000
BAFF (pg/mL)
Spearman-R: 0.3259
P-value: 0.0429 (*)
C.
HIV- CVD+
Total Plaque Volume (mm3)
2500
2000
1500
1000
500
0
0
500
1000
1500
2000
2500
BAFF (pg/mL)
Spearman R: 0.3903
P-value: 0.06
